# Retention in clinic versus retention in care during the first year of HIV care in Nairobi, Kenya: a prospective cohort study

**DOI:** 10.1002/jia2.25196

**Published:** 2018-11-29

**Authors:** Mia L van der Kop, Patrick I Nagide, Lehana Thabane, Lawrence Gelmon, Lennie B Kyomuhangi, Bonface Abunah, Anna Mia Ekström, Richard T Lester

**Affiliations:** ^1^ Department of Public Health Sciences/Global Health (IHCAR) Karolinska Institutet Stockholm Sweden; ^2^ Department of Medicine University of British Columbia Vancouver BC Canada; ^3^ Amref Health Africa Nairobi Kenya; ^4^ Department of Health Research Methods, Evidence and Impact McMaster University Hamilton ON Canada; ^5^ Biostatistics Unit St Joseph's Healthcare—Hamilton Hamilton ON Canada; ^6^ Department of Medical Microbiology University of Manitoba Winnipeg MB Canada; ^7^ Jhpiego Johns Hopkins University Affiliate Baltimore MD USA; ^8^ Department of Infectious Diseases Karolinska University Hospital Stockholm Sweden

**Keywords:** HIV care continuum, cohort studies, informal settlements, HIV/AIDS, patient retention, Kenya

## Abstract

**Introduction:**

When measuring the success of HIV programmes to retain patients in care, few studies distinguish between retention in clinic (individual returns to the same clinic) and retention in care (individual is active in care at initial site or elsewhere). The objectives of this study were to quantify retention in clinic versus retention in care and determine risk factors associated with attrition from care in low‐income settings in Nairobi, Kenya.

**Methods:**

Between April 2013 and June 2015, adults testing positive for HIV were recruited at two comprehensive care clinics in informal urban settlements. Participants were followed from the time of a positive HIV test for up to 14 months. Participants who did not return to the clinic for their 12‐month appointment between 10 and 14 months after their baseline visit were traced by telephone or community outreach to determine whether they were still receiving HIV care. We used generalized linear regression to determine the association between clinical and socio‐demographic factors and attrition from care at 12 months.

**Results:**

Of the 1068 individuals screened for study participation, 775 individuals newly presenting to HIV care were included in this study. Between 10 and 14 months, 486 participants (62.7%, 95% confidence intervals [CI], 59.2% to 66.1%) returned to the clinic for their 12‐month appointment (retained in clinic). After telephone tracing and community outreach, an additional 123 of 289 participants were found to be active in care at other HIV clinics (42.6%, 95% CI, 36.8% to 48.5%). Overall, 609 (78.6%, 95% CI, 75.7% to 81.5%) participants were retained in care at any HIV clinic at 12 months. Participants in higher baseline CD4 count categories were more likely to be retained than those whose baseline CD4 count was <200 cells/mm^3^.

**Conclusions:**

Retention in clinic substantially underestimated retention in care 12 months after presenting to care in this high‐prevalence and low‐income urban setting. Improved systems to track patients between clinics are required to accurately estimate retention in care in resource‐limited settings. Although the proportion of patients retained in care was greater than expected, interventions to improve retention in care are needed to meet global targets to end the AIDS epidemic.

## 1 Introduction

Patient retention in care is fundamental to meeting the UNAIDS 90‐90‐90 targets by 2020: to have 90% of people living with HIV know their status; 90% of those who know their status on antiretroviral therapy (ART); and 90% of those on ART having undetectable viral loads 1. Despite the importance of retention in care, most studies derive estimates of *retention in care* using *retention in clinic* as a proxy measure. The increased urbanization together with the decentralization of HIV care in sub‐Saharan Africa (SSA) has meant individuals with HIV have more options as to where they seek their care, therefore, retention in clinic and retention in care are likely to differ.

Similar to Geng *et al*. 2, we consider retention from a patient‐based perspective, where those with HIV are considered retained in care if they remain active in care, regardless of whether this care is received from a different clinic to the one in which they were originally enrolled. This is opposed to retention in clinic, which considers retention from a clinic perspective, in which patients are considered retained if they return to the same clinic at a particular time point. Tracing studies have the advantage of providing more accurate estimates of retention in care; however, few studies have had a tracing component, or have only traced a sample of those who did not return to the clinic 2.

Here, to provide an accurate estimate of retention in care at one year, regardless of ART status, we conducted a prospective cohort study where we traced all participants who did not return to the clinic at which they were enrolled. Our objectives were to quantify the proportion of individuals retained in clinic versus retained in care at 12 months and to determine risk factors for attrition from HIV care at 12 months.

## 2 Methods

### 2.1 Study design

During recruitment for a randomized controlled trial (RCT) in Kenya to test the effectiveness of a text‐messaging intervention to improve retention in early HIV care 3, we enrolled HIV‐positive participants for this supplementary cohort study. In this study, our population consisted of trial participants and cohort study participants, who were followed for up to 14 months (12 months plus or minus a two‐month window). The rationale for enrolling participants in supplementary cohort study alongside the RCT was to create a more generalizable cohort than that which would have been created if we had studied the trial cohort alone. As specified in the published trial protocol, the supplementary cohort study was established to enable us to examine patient retention during the first year of HIV care 4.

### 2.2 Study setting and participants

Between 4 April 2013 and 4 June 2015, participants were recruited from the Kibera Community Health Centre, an Amref Health Africa clinic located in a large informal settlement in Nairobi. At this comprehensive care clinic, there are no direct patient costs for HIV care and treatment. The population the clinic serves lacks or has minimal access to services such as public education, clean water, modern sanitation and other basic public services. HIV prevalence among adults is estimated at 12% 5, about twice the average national prevalence 6. In February 2014, recruitment began at a second comprehensive care clinic, the Baba Dogo Health Centre, situated in another large informal settlement in Nairobi's Eastlands area and operated by the University of Manitoba/Partners for Health and Development in Africa (PHDA).

For this study, we included both patients with mobile phone access who were enrolled in the interventional RCT and those without access to provide a more comprehensive cohort related to retention in care. At each site, clinical staff members introduced potential participants to a research nurse, who completed an eligibility assessment. Patients were invited to enrol in the supplementary cohort study if they did not fulfil the trial's phone‐related eligibility criteria (because they did not have mobile phone access or could not text message and did not have somebody who could text message on their behalf). Patients were eligible to participate in the cohort study if they tested positive for HIV (potential participants were given one week to decide to enrol or not), were at least 18 years of age, and were willing to provide informed consent. Both ART‐ineligible and ART‐eligible patients were eligible for the study. Patients previously assessed for ART eligibility, with prior ART exposure, or on ART were excluded. Women known to be pregnant were also excluded.

Baseline laboratory testing included two rapid HIV tests. The first was Alere Determine HIV‐1/2 (https://www.alere.com/en/home/product-details/determine-hiv-1-2.html). Uni‐Gold (http://www.trinitybiotech.com/area/uni-gold/) was used as a confirmatory test. CD4 counts were measured at the baseline visit and every six months thereafter. From the beginning of recruitment in April 2013 until 2014, the clinics used a threshold CD4 cell count of ≤350 cells/mm^3^ to determine ART eligibility. In 2014, the clinics adopted the WHO recommendation of using a CD4 cell count of ≤500 cells/mm^3^ to determine ART eligibility. The Baba Dogo clinic implemented the new guidelines in August 2014, and the Kibera clinic in September 2014.

**Figure 1:**
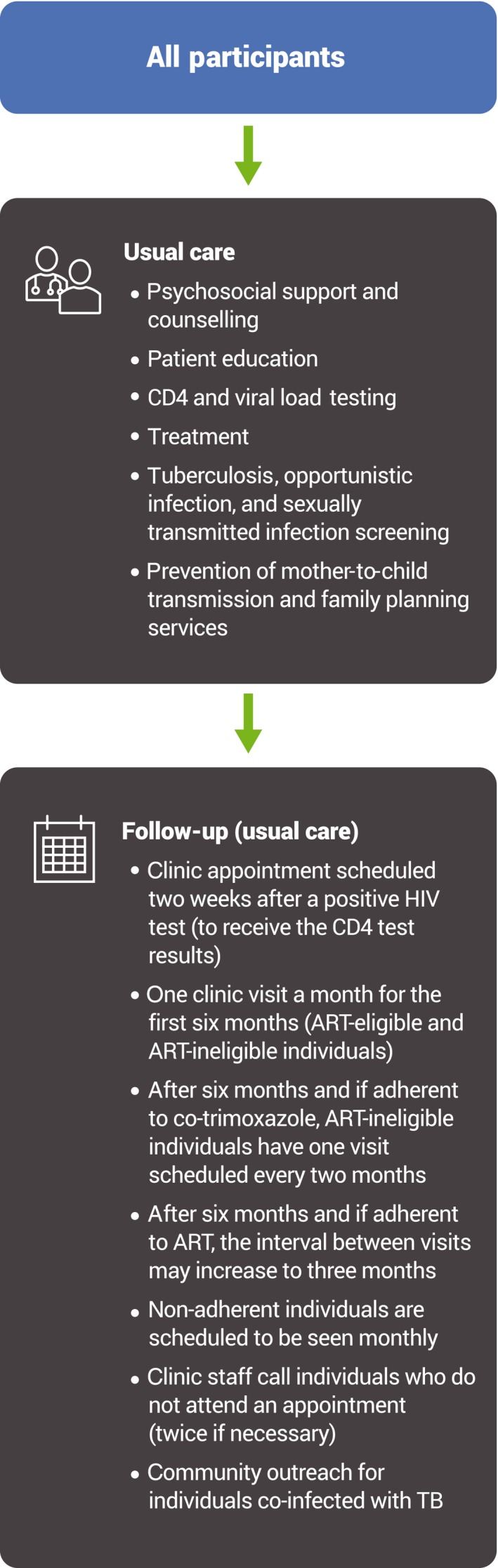
Usual care

All participants in the cohort study received standard of care (Figure 1). As part of standard care, patients are called up to two times if they do not attend a scheduled clinic appointment. Community tracing is not a part of routine care except in cases in which patients are co‐infected with tuberculosis; mothers are enrolled in a prevention of mother‐to‐child transmission programme; or for children or adolescents under the care of a parent or guardian. Participants were followed up for a maximum of 14 months, at which point tracing activities began. Apart from end‐of‐study tracing, research activities were minimized to avoid influencing usual clinical care, for example, there were no additional research visits. Tracing was conducted if participants did not attend their 12‐month follow‐up appointment within 10 to 14 months after their first visit. First, a research nurse telephoned participants to ascertain their care status (if a participant could not be reached, the nurses tried alternate contact phone numbers, which had been collected at enrolment). If contact was not made by telephone, then experienced community health workers traced participants in their communities to determine their status, for example, informally transferred care and active in care, confirmed as defaulting from care, etc. Follow‐up concluded on 22 September 2016.

### 2.3 Outcomes

#### 2.3.1 Retention in clinic

Retention in clinic was defined as the proportion of participants who attended their 12‐month appointment after testing HIV positive at the clinic of enrolment within a 10‐ to 14‐month window, regardless of ART status.

#### 2.3.2 Retention in care

Retention in care was defined as the proportion of participants retained in care 12 months following a positive HIV test, measured by whether the participant attended a follow‐up appointment at any HIV treatment clinic within 10 to 14 months after their first visit, regardless of ART status. Participants who did not return to the clinic of enrolment but who were confirmed active in care elsewhere were considered retained in care.

$$\begin{array}{c} \text{retention in care at 12 months} \end{array}=\frac{\text{participants retained in clinic + participants confirmed active in care elsewhere}}{\text{all enrolled study participants}}$$

#### 2.3.3 Quantitative variables

We selected variables based on a literature review of factors found to be associated with attrition from HIV care. Demographic and clinical variables included sex 7, 8 (male or female), age 7, 8, education (no secondary vs. some secondary), previous HIV diagnosis, CD4 count 7, 8 and ART eligibility at baseline <200; 200 to 349; 350 to 499; ≥500 cells/mm^3^). We also explored travel time to clinic 7 (<60 minutes vs. ≥60 minutes), alcohol use (hazardous drinking, non‐hazardous drinking (AUDIT‐C)) 9 and perceived social support (someone to turn to none/a little/some of the time vs. most/all of the time) as potential risk factors for attrition. Clinic attended (Baba Dogo vs. Kibera) was also considered. We investigated an interactive effect between travel time to clinic and sex based on results from a previous study on retention in care in Kenya 7.

### 2.4 Data sources

At the baseline visit, the research nurses administered a questionnaire in the participant's language of choice, English or Kiswahili. The questionnaire collected information on demographic characteristics and HIV testing history. Blood was drawn at the baseline visit for laboratory CD4 testing. Clinic visit data were collected on study‐specific forms that clinicians filled in at each visit. Clinical records were also used to establish whether a participant had formally transferred their care. At 14 months, a tracing form was completed for each participant. Information was collected on the status of participants (e.g. death, confirmed as defaulting from care, active in care elsewhere) who did not return to the clinic for their 12‐month appointment and the type of tracing that was undertaken, that is, telephone or community outreach. Data were entered in Microsoft Access weekly. Verification procedures included cross‐checking data with original forms and clinical records, as well as range and consistency checks.

### 2.5 Study size

A conservative rule is that logistic regression models should have 10 outcome events per predictor variable to build stable models 10. Based on an estimate of the percentage retained in care at 12 months of 80% 3 and 775 participants (700 trial participants and 75 participants in the supplementary cohort study), it was expected that we would have 155 attrition events in this cohort. This is considered adequate to build stable models with the 11 selected factors.

### 2.6 Statistical methods

We determined the frequency of participants lost to care and used descriptive statistics to summarize baseline characteristics of the study population. Generalized linear regression with a log link and binomial distribution was used to test whether the selected factors were associated with attrition from care (rather than attrition from clinic). First, bivariate analyses were performed to assess the crude association between each factor and the outcome. Then, all variables were included in a multivariable model. Interaction between variables and tests of linear assumption (for variables with multiple categories) were examined using nested models and the likelihood ratio test. Results are presented as risk ratios (RR) and adjusted RRs (ARR) with corresponding 95% confidence intervals (CI). Analyses were performed using Stata version 12 (Statacorp, College Station, TX).

### 2.7 Ethics

Ethics approval was obtained from the University of British Columbia's Clinical Research Ethics Board (H12‐00563) and Amref Health Africa's Ethics and Scientific Review Committee (P40/12). Participants provided written informed consent to participate; except for illiterate individuals, who provided consent with a thumb print in the presence of a literate witness. Participants were informed that they were free to withdraw from the study at any time.

## 3 Results

### 3.1 Participants

**Figure 2:**
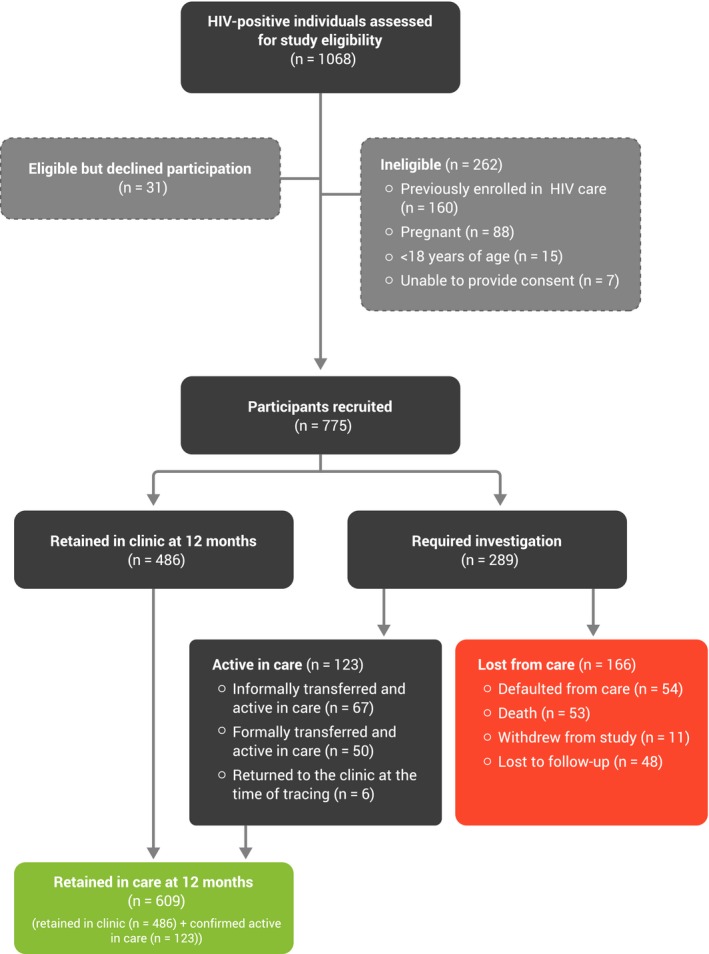
Participant recruitment flow diagram

Between 4 April 2013 and 4 June 2015, we screened 1068 individuals with HIV for study participation (Figure 2). Of those, 24.5% (n = 262) were ineligible for the study. Reasons for ineligibility included previous enrolment in HIV care (n = 160, 61.1%) and pregnancy (n = 88, 33.6%). Of those screened, 2.9% (n = 31) were eligible but declined participation (Figure 2). Of the 775 participants who were recruited, 700 participated in an RCT and were included in this cohort study and 75 participated in the cohort study only.

### 3.2 Baseline characteristics

Baseline demographic and clinical characteristics for both retained and non‐retained participants are presented in Table 1. Overall, the mean age of the cohort was 34 years (SD, 9.83) and 60.6% (n = 470) of the cohort was female. The median CD4 cell count was 302 cells/mm^3^ (quartile 1 (Q1) 148 to quartile 3 (Q3) 462) and 58.6% (n = 454) had been previously diagnosed with HIV before their positive HIV test result at the time of enrolment.

**Table 1:** Baseline characteristics

Variable	Attrition from care at 12 months (n = 166)	Retained in care at 12 months (n = 609)
Sex		
Male	74 (24.3)	231 (75.7)
Female	92 (19.6)	378 (80.4)
Age (years)		
Mean (SD)	34.7 (10.8)	33.7 (9.5)
<30	61 (20.8)	232 (79.2)
30 to 39	59 (20.3)	232 (79.7)
40 to 49	27 (24.6)	98 (78.4)
≥50	19 (28.8)	47 (71.2)
Education		
No secondary school	120 (23.0)	401 (77.0)
Some secondary school	46 (18.1)	208 (81.9)
Previous HIV diagnosis		
No	72 (22.4)	249 (77.6)
Yes	94 (20.7)	360 (79.3)
CD4		
Median (IQR) (cells/mm^3^)	252 (84 to 450)	314 (168 to 464)
<200	66 (26.7)	181 (73.3)
200 to 349	33 (15.7)	177 (84.3)
350 to 499	23 (16.3)	118 (83.7)
≥500	32 (20.7)	123 (79.4)
Missing	12 (54.5)	10 (45.5)
ART eligibility at baseline		
Ineligible	43 (19.6)	176 (80.4)
Eligible	111 (20.8)	423 (78.2)
Missing	12 (54.5)	10 (45.5)
Clinic		
Kibera	129 (23.3)	425 (76.7)
Baba Dogo	37 (16.7)	184 (83.3)
Trial participant		
No	28 (37.3)	47 (62.7)
Yes	138 (19.7)	562 (80.3)
Social support		
None/a little/some of the time	35 (22.6)	120 (77.4)
Most/all of the time	131 (21.1)	489 (78.9)
Travel time to clinic		
<60 minutes	97 (16.1)	507 (83.9)
≥60 minutes	23 (19.2)	97 (80.8)
Missing	1 (16.7)	5 (83.3)
Alcohol use		
Non‐heavy/hazardous drinking	128 (20.3)	503 (79.7)
Heavy/hazardous drinking	38 (26.4)	106 (73.6)

Values are numbers (percentages).

SD, standard deviation; IQR, interquartile range.

### 3.3 Follow‐up

Participants were followed up for a median of 55 weeks (Q1 to Q3, 51 to 60 weeks), during which they attended a median of 10 appointments (Q1 to Q3, 6 to 12 appointments). During the study, 615 of the 775 participants initiated ART at the study clinics, and 11 participants withdrew. We investigated the outcomes of 289 participants who did not return to the clinic for their 12‐month appointment between 10 and 14 months. Where study and clinical records did not reveal the participant's final status (n = 153), 136 participants’ outcomes were determined through telephone tracing and 17 participants required further community tracing to assess their final status. The median time from enrolment to last visit for those who were not retained in clinic was 6 weeks (Q1 to Q3, 1 to 20 weeks).

### 3.4 Retention in clinic versus retention in care

Overall, 486 of the 775 participants (62.7%, 95% CI, 59.2% to 66.1%) were retained in clinic at 12 months (returned to the clinic for their 12‐month visit between 10 and 14 months), whereas 609 of the 775 (78.6%, 95% CI, 75.7% to 81.5%) were retained in care at 12 months (returned to the clinic for their 12‐month visit or were confirmed active in care elsewhere) (Figure 2). Those who withdrew from the study were included in the denominator (n = 775) and subtracted from the numerator, that is, considered lost to care. Of the 123 people who were retained in care but did not return to the original clinic within the 10‐ to 14‐month timeframe, 67 had informally transferred care and were confirmed active at their new clinic; 50 had formally transferred care and were confirmed to be active in care; and 6 returned to the clinic at the time of tracing. Overall, 155 participants were lost from care: 54/775 (7.0%) were confirmed as defaulting from care; 53 (6.8%) had died and 48 (6.2%) were lost to follow‐up, that is, we could not determine their status (active in care, defaulted from care, or death) through telephone or community tracing (Figure 2). The median time from first to last visit was 5 weeks (Q1 to Q3, 1 to 12 weeks) for those who had died; 5 weeks (Q1 to Q3, 0 to 18 weeks) for those who had informally transferred; 10.5 weeks (Q1 to Q3, 2 to 21 weeks) for those who had formally transferred; and 2.5 weeks (Q1 to Q3, 0 to 13.5 weeks) for those who were LTFU.

### 3.5 Factors associated with attrition from care

In the crude analysis, presenting at the Baba Dogo clinic and being a trial versus a cohort study‐only participant were associated with a reduced risk of attrition (Table 2). In the multivariable model, being a trial participant remained strongly associated with a reduced risk of attrition ((ARR) 0.52 95% CI, 0.37 to 0.73). Compared to the baseline CD4 count category of 200 cells/mm^3^, participants in higher CD4 count categories had a reduced risk of attrition (ARR 0.59 (95% CI, 0.40 to 0.85) for those in the 200‐349 cells/mm^3^ CD4 count category and ARR 0.61 (95% CI, 0.35 to 1.08) for those in the 350‐499 cells/mm^3^ CD4 count category). An interactive effect between sex and travel time to clinic was not found.

**Table 2:** Outcomes. Univariable and multivariable analysis of variables associated with 12‐month attrition from care

Variable	Crude risk ratios		Adjusted risk ratios^b^	
	RR	95% CI	RR	95% CI
CD4 (cells/mm^3^)^a^				
<200	Reference		Reference	
200 to 349	0.59	0.40 to 0.86	0.59	0.40 to 0.85
350 to 499	0.61	0.40 to 0.94	0.61	0.35 to 1.08
≥500	0.77	0.53 to 1.12	0.77	0.33 to 1.79
ART‐eligible at baseline^a^	1.06	0.77 to 1.45	0.99	0.47 to 2.09
Trial versus cohort study‐only participant	0.53	0.38 to 0.73	0.52	0.37 to 0.73
Male	1.24	0.95 to 1.62	1.25	0.93 to 1.68
Presenting at the Baba Dogo Clinic	0.72	0.52 to 1.00	0.72	0.52 to 1.01
Age (per year increase)	1.01	0.99 to 1.02	0.99	0.98 to 1.01
No secondary education	1.27	0.94 to 1.73	1.11	0.80 to 1.53
Previous HIV diagnosis	0.92	0.70 to 1.21	0.97	0.73 to 1.28
Social support (All/most vs. some/little/none of the time)	1.07	0.77 to 1.48	1.06	0.75 to 1.49
Time to clinic	0.95	0.78 to 1.16	0.91	0.72 to 1.16
Hazardous drinking	1.30	0.95 to 1.78	1.22	0.88 to 1.68

RR, risk ratio; CI, confidence interval.

^a^Missing data for 22 participants; ^b^adjusted model based on 753 participants (excludes 22 participants with missing CD4 data).

## 4 Discussion

This study showed that *retention in care* is substantially greater than *retention in clinic* during the first year of HIV care and highlights the importance of obtaining more accurate estimates of retention in care. At 12 months, 63% (95% CI, 59.2% to 66.1%) of participants returned to the clinic for an appointment (*retained in clinic*), whereas 79% (95% CI, 75.7% to 81.5%) of participants were active in HIV care at the site of enrolment or elsewhere (*retained in care*). Retention in care was 16% greater than retention in clinic. Having a higher baseline CD4 count and participating in the trial versus the cohort study only were associated with a reduced risk of attrition. Most of those who did not return to the same clinic but were retained in care had informally, versus formally, transferred their care to another clinic. Of those who were lost to care, defaulting from care and death contributed almost equally to attrition.

A Ugandan study examined retention in care among those on ART using a sampling‐based approach, in which a tracker community traced a sample of 128 participants out of 829 who did not return to the clinic to estimate retention in care 11. In the Ugandan study, participants were traced to ascertain their vital status 11, and not to determine specifically whether they were active in HIV care. Estimates of retention in care at 12 months in the Ugandan study were based on one of two scenarios: assuming all patients who were determined to be alive in the sample were retained in care and extrapolating this to estimate retention in care, which increased the estimate of retention in care to 90.9% from 82.3% who were retained in clinic; or alternatively, assuming all patients who were found alive were no longer in HIV care, which increased the estimate of retention in care to 85.8% from 82.3% 11. The magnitude of difference between retention in clinic and retention in care is much larger in our study than even in Geng *et al*.'s most optimistic scenario (in which all patients found alive were presumed to be active in HIV care). A possible reason for this is the difference in the study setting. The Ugandan study took place in a rural area, whereas our study took place in Nairobi, where people living with HIV have more HIV care options. In both areas where our study took place, there are many other clinics that provide HIV care. In addition, slum populations in Nairobi are highly transient, which may have led to more transfers of care, both formal and informal.

Systematic reviews and meta‐analyses of retention in care estimate retention in SSA for those in ART programmes at 75.0% 12 to 80.2% 13. Among pre‐ART patients, retention in care is much lower 14, estimated at 54.2% 15. In our study, most participants were ART‐eligible at baseline; however, similar to other studies, retention in care was lower among those not eligible for ART. Retention in clinic for those ineligible for ART was 55.0%. This increased to 66.9% when those active in care elsewhere were considered. During the study, treatment guidelines changed from a CD4 threshold of 350 cells/mm^3^ to 500 cells/mm^3^, and most of the participants (n = 615/775, 79.4%) initiated ART at the study clinics at some point within the study period. The World Health Organization now recommends at “treat all” strategy, in which all people with HIV are eligible for ART, regardless of CD4 count. It remains to be seen whether the change in ART guidelines to a “treat all” policy will improve retention in care, or whether these individuals eligible for ART earlier in the course of infection will face different barriers to retention.

Individual studies, and consequently, reviews and meta‐analyses, estimate retention in care base their estimates on *retention in clinic* rather *retention in care*. Few studies report transfers between clinics and do not confirm whether participants were active in care in clinics other than the ones at which they had originally enrolled at specific time points 15. The lack of reporting on transfers and the absence of tracing activities in studies estimating retention have led many authors to conclude that attrition is likely overestimated 2, 11, 15. Our study, together with Geng *et al*.'s study 11 and a study from South Africa 16, confirms that retention in clinic underestimates retention in care in some settings. In the case of our study population, retention in care was 25% greater than retention in clinic at 12 months. The South African study estimated retention in care among those who had initiated ART. They concluded that after accounting for transfers of care, 6‐year retention increased from 29.1% (95% CI, 28.7% to 29.5%) to 63.3% (95% CI, 62.9% to 63.7%) 16. HIV programmes with stronger monitoring and evaluation capabilities may have more accurate reporting of retention in clinic or care, and those with poorer tracking and resources may have different, even lower, indicators of patient retention. In such cases, strengthening patient tracking and support, perhaps through digital systems, patient tracers, or better information systems, may improve clinic visit data and provide opportunities to support engagement in care.

In this study, a higher CD4 was associated with a reduced risk of attrition from care, which is similar to findings from other studies 7, 8, 15. Those with lower CD4 cell counts are more likely to be lost to programme because of increased mortality 17. Similar to Mugglin *et al*. 15, we also found that men were more likely to be lost to care, although in our findings, the confidence interval included one. A strong factor related to retention in our study was whether a participant had been enrolled in the trial or the cohort study only. As the intervention tested in the trial had no effect on retention 3, and there were no procedural differences (clinical or study‐related) between the trial and cohort participants, differences between the trial and cohort participants may have given rise to their differing risk of attrition. Participants who did not own or have access to a mobile phone, or who could not text message or have somebody text message on their behalf, were ineligible for the trial but eligible to participate in the cohort study. Lack of access to a mobile phone and an inability to text or have somebody who could text on their behalf may be a proxy indicator for level of education, social support and socio‐economic status (SES). Lower SES and less social support have been found to be associated with attrition 2, 15, which may explain why trial participants were more likely to be retained than cohort participants.

Ours is the first study to trace all participants who did not return the clinic for their 12‐month appointment specifically to determine whether they were active in HIV care. Our high participation rate, with 2.9% of those eligible having declined participation, and low LTFU (6.2%), minimize selection bias 18. While our study has strong internal validity, the two clinics involved in the study were located in informal urban settlements, so our findings might not be generalizable to rural areas where there are fewer HIV services, in higher income settings, or among less transient populations. Another limitation of this study is that our measure of retention in care was restricted to being active in care at 12 months, which does not provide information about adherence to care during those 12 months. It is possible that some participants who had a 12‐month visit as per the study definition did not attend all routine visits, which may have led to suboptimal treatment outcomes. Finally, the final status (e.g. defaulted from care, informal transfer of care) of participants who were traced was reported by the participants themselves, which may have been influenced by social desirability bias.

## 5 Conclusions

We observed a substantial gap between retention in clinic and retention in care, indicating that retention in clinic may be a poor proxy for retention in care in some settings. Methods that track patient care between clinics, including silent transfers, are required to more accurately estimate retention in care. Our findings are positive in that a greater proportion of people living with HIV were retained in care than was expected; however, our estimate of retention in care falls short of the level of retention required to fulfil the UNAIDS 90‐90‐90 targets, necessitating new interventions to better retain people in HIV care.

## Competing Interests

None of the authors declared conflicts of interest.

## Authors’ Contributions

RTL and MVDK conceived the study. MVDK designed the study with input from RTL, LT, LG, LBK and AME. PIN and BA acquired the data. LT provided statistical advice. MVDK performed the statistical analyses and drafted the manuscript. PIN, LT, LG, LBK, BA, AME and RTL revised the manuscript for important intellectual content. All authors approved the submitted version of the manuscript.
